# A unique method for estimating the reliability learning curve of optic nerve sheath diameter ultrasound measurement

**DOI:** 10.1186/s13089-016-0044-x

**Published:** 2016-08-08

**Authors:** Frederick A. Zeiler, Markus T. Ziesmann, Patrick Goeres, Bertram Unger, Jason Park, Dimitrios Karakitsos, Michael Blaivas, Ashley Vergis, Lawrence M. Gillman

**Affiliations:** 1 Departments of Surgery, University of Manitoba, 409 Tache Avenue, Winnipeg, MB R2H 2A6 Canada; 2 Undergraduate Medical Education, University of Manitoba, Winnipeg, MB Canada; 3 Medical Education, University of Manitoba, Winnipeg, MB Canada; 4 Departments of Internal Medicine, University of South Carolina, School of Medicine, Columbia, SC USA; 5 Departments of Internal Medicine and Emergency Medicine, University of South Carolina, School of Medicine, Columbia, SC USA

**Keywords:** Point of Care ultrasound, Optic nerve sheath diameter, Ultrasound, Intracranial pressure, Education, Learning curve

## Abstract

**Background:**

Optic nerve sheath diameter (ONSD) measurement using ultrasound has been proposed as a rapid, non-invasive, point of care technique to estimate intra-cranial pressure (ICP). Ultrasonic measurement of the optic nerve sheath can be quite challenging and there is limited literature surrounding learning curves for this technique. We attempted to develop a method to estimate the reliability learning curve for ONSD measurement utilizing a unique definition of reliability: a plateau in within-subject variability with unchanged between-subject variability.

**Methods:**

As part of a previously published study, a single operator measured the ONSD in 120 healthy volunteers over a 6-month period. Utilizing the assumption that the four measurements made on each subject during this study should be equal, the relationship of within-subject variance was described using a quadratic-plateau model as assessed by segmental polynomial (knot) regression.

**Results:**

Segmental polynomial (knot) regression revealed a plateau in within-subject variance after the 21st subject. However, there was no difference in overall mean values [3.69 vs 3.68 mm (*p* = 0.884)] or between-subject variance [14.49 vs 11.92 (*p* = 0.54)] above or below this cutoff.

**Conclusions:**

This study suggests a significant finite learning curve associated with ONSD measurements. It also offers a unique method of calculating the learning curve associated with ONSD measurement.

## Background

In head injured patients, elevated intra-cranial pressure (ICP) is associated with worse outcomes [1]. One of the fundamental tenants of management involves identifying those with elevated ICP and intervening to lessen and control this pressure [2]. Optic nerve sheath diameter (ONSD) measurement using ultrasound has been proposed as a rapid, non-invasive, point of care technique to estimate ICP [3]. This has been suggested as a screening tool for, or even an alternative to, invasive ICP measurement. Traditionally ICP is measured via a surgically inserted, intracranial (subdural, intra-parenchymal or intra-ventricular) pressure monitor. While this is a fairly common procedure it carries a finite but not insignificant rate of complications (most notably intracranial hemorrhage and infection) [4] and is for the most part limited to centres with neurosurgical capabilities. Given the growing use of ultrasound in the emergency department, critical care units and even in the field, point-of-care estimation of ICP using ultrasound is appealing as it can be performed in a wide variety of locations, is easily repeatable and does not require transport of the patient or large diagnostic equipment (such as CT scanners). Its utility has been substantiated by a number of small studies in diverse populations including traumatic brain injury, mass lesion, infection and post transplant reperfusion injury [5–9].

The technique, however, can be quite challenging. It requires the measurement of an often moving, sub-centimeter structure, estimated to the nearest 0.1 of a millimeter, stretching both the limits of the operator and the resolution of the ultrasound unit. Significant inter-rater variance early in the learning curve is a potential concern that could affect the utility of this approach in more novice users. The literature surrounding learning curves for point of care ultrasound in general is limited [10] and even more so for the specific technique of ONSD measurement [11]. Here we attempt to employ a unique statistical method for estimating the potential learning curve for ONSD measurement.

## Methods

### Ultrasound technique

As part of a previously published study [12] a single trained operator (LG) measured the ONSD in 120 healthy volunteers over a 6-month period. This operator was trained in this technique during a World Interactive Network Focused On Critical Ultrasound (WINFOCUS) ultrasound course and has a number of previous publications in this area [12–14]. All subjects signed a consent form approved by the University of Manitoba Health Research Ethics Board (H2013:199).

**Fig. 1:**
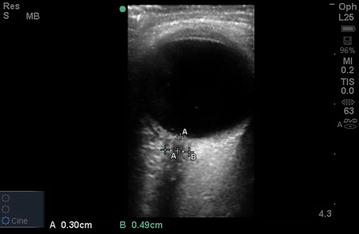
Sample image of ultrasonic ONSD measurement (measurement “*B*”), measured 3 mm behind the optic nerve head (measurement “*A*”)

Subjects were placed in the supine position and images were obtained through a closed eyelid. A 13–6 MHz linear array ultrasound transducer (L25x transducer with a Sonosite M-Turbo Ultrasound Machine, SonoSite Inc, Bothell, WA) was used with the ophthalmic preset thus minimizing the power and acoustic output of the ultrasound system. The probe was placed gently on the upper eyelid. The ONSD was measured in a direction perpendicular to the axis of the nerve, in each eye, in the horizontal and sagittal plane, 3 mm behind the optic nerve head (Fig. 1), for a total of four measurements per subject.

### Method for assessing the learning curve

For the sake of the learning curve analysis it was assumed that the four measurements (left, right, horizontal, sagittal) in each patient should be identical and any differences in these values are the result of measurement error.

The relationship of within-subject variance was described using a quadratic–plateau model as assessed by segmental polynomial regression [15] to determine how many subject measurements were needed before the operator achieved a stable within-subject variance. Segmental polynomial regression, or knot regression, allows the opportunity to fit multiple regression lines to different portions of a dataset, which then join at a “knot” value which represents the intersection of these two regression lines (in this case the plateau of the learning curve). In this specific variant of segmental polynomial regression, the quadratic-plateau model, a quadratic curve is fit to the early data and a flat line (plateau) to the later data. This model is able to predict the exact point of plateau in the learning curve as it describes how many subjects were needed before the ultrasonographer achieved a stable within-subject variance.

### Assessing data before and after the “knot”

To ensure there was no inherent differences in the data other than the plateau of within-subject variance before and after the “knot” variable as described above, the means of the early (subjects before the “knot” or plateau) versus late (subjects after the “knot”) measurements were compared using Welch Two Sample *t* tests. As well, the homogeneity of the between-subject variance was examined for early versus late measurements utilizing Levene’s test for homogeneity of variance.

Analysis was completed using both the Statistical Analysis Software version 9.3 (SAS Institute, Cary, NC) and R Statistical Library (R Core Team, 2013).

## Results

Subjects included 65 males and 55 females. Mean age was 29.3 (range 18–65). Overall mean ONSD measurement was 3.68 (95 % CI 2.85–4.40) [12]. Segmental polynomial (knot) regression revealed a plateau in within-subject variance, var(*I*), at subject index (*I*) 21 (Fig. 1). Below the knot (Subject 21), the best-fit equation is:

$$ {\text{var}}(I) = 19.73 - 1.614I + 0.03843I^{2} $$

Above the knot, the best-fit equation is simply the plateau value:

$$ {\text{var}}(I) = 2.78 $$

Based on the segmental polynomial regression and the plateau of within-subject variance at subject 21 we utilized this as the cut-off for defining early (subjects before the “knot” or plateau) vs late (subjects after the “knot”) measurements. The mean measurements for each subject were compared between the early and late groups using Welch Two Sample *t* tests (Table 1) with no difference seen. In addition, utilizing Levene’s test for homogeneity, the between-subject variance was compared in these two groups revealing no difference between early and late groups [14.49 (early) vs 11.92 (late) (*p* = 0.54)]. For males and females, respectively, it was 12.64 (early) vs 9.48 (late) (*p* = 0.33) and 12.50 (early) vs 11.71 (late) (*p* = 0.95).

**Table 1:** Welch two sample *t* test comparing mean of early versus late mean ONSD measurements using subject 21 as a cutoff

	Early ONSD mean (mm)	Late ONSD mean (mm)	*p* value
Overall	3.69	3.68	0.884
Males	3.88	3.78	0.454
Females	3.52	3.55	0.869

## Discussion

Determining the learning curve associated with point of care ultrasound has been investigated in multiple studies across a number of techniques. The most commonly studied technique is the focused assessment with sonography in trauma (FAST) exam [10]. Estimates for the learning curve associated with this technique have been extremely variable, however, and range from 10 [16, 17] to 200 studies [18] and even up to 500 scans [19]. The heterogeneity in these results likely results from the fact that there is no clear, accepted definition of what is an acceptable performance. The majority of these studies utilized clinical endpoints (correct identification of free fluid) to define the learning curve with varying thresholds of acceptable results [10, 16–18, 20–23], while only a single study utilized an assessment of image quality and technique [24].

To our knowledge, only a single study to date has commented on the learning curve associated with ONSD measurement. In their 2007 study, Tayal et al. [11] concluded that this technique can be learned relatively quickly with a learning curve of between 10 and 25 scans depending on previous ultrasound experience. However, the origin of these estimates is not clearly discussed within the manuscript and in discussion with the senior author of this paper, these figures were estimated based on qualitative observation and not based on objective measures (personal communication).

As in other forms of point of care sonography, one of the challenges associated with determining a learning curve involves the definition of acceptable performance. This is even more so an issue for ONSD measurement as it requires a quantitative assessment of a specific structure that is inherently difficult to measure making comparison to a control or reference value challenging. For example, in comparison with the work done in FAST one could use clinical outcomes and prediction of elevated ICP as a surrogate of competency but this does not determine if the numeric measurement of ONSD is accurate. Alternatively, one could use an assessment tool of ONSD image quality and generation but currently as apposed to FAST [25], no such validated tool exists. One could use comparison of results to another imaging technique (MRI or CT scan). While this has been used to assess the utility of ultrasound ONSD measurement [6] to date this has not been used to assess learning curves. One could also compare to the results of an expert but again this requires the definition of an expert. Finally, one could use a model with known ONSD measurements as was recently developed by our group [13, 14]. While measurements in the model tend to be easier than real life and may underestimate the learning curve-crisper borders to the simulated optic nerve sheath, and no eye movement to contend with-of the options available, it certainly seems the most feasible and reproducible and an area requiring further study.

**Fig. 2:**
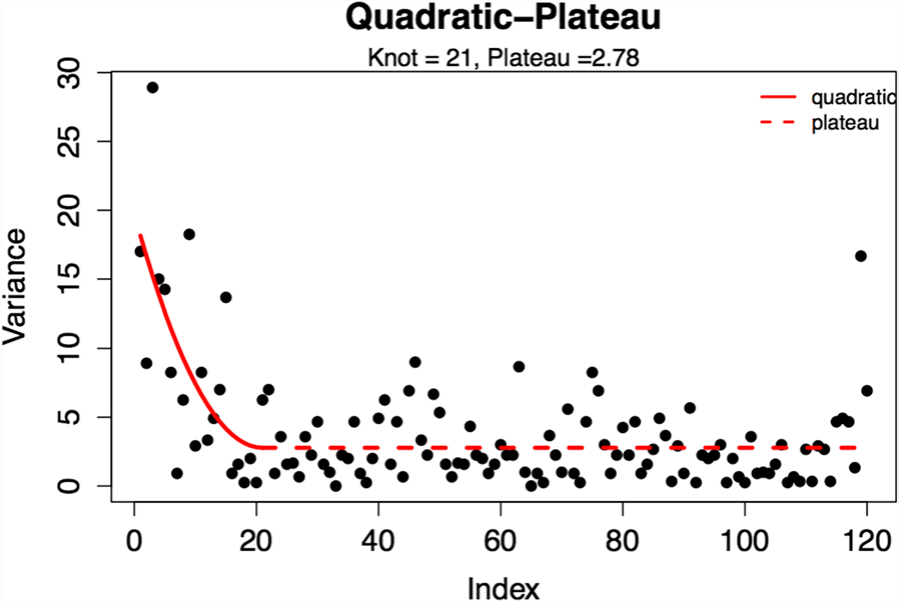
Segmental polynomial (knot) regression of within-subject variance showing a plateau (knot) at subject 21

One important dimension in proficiency with this skill is the ability to making reliable measurements. In this study, we utilized a unique definition of reliability. First, by assuming the presence of an internal control between each measurement in each eye and assuming any error/variance between these measurements is due measurement error. There are a number of recent ultrasound studies that give credence to this assumption by suggesting there may be no difference between right and left ONSD measurements [26–28]. In addition, since the variance did indeed plateau over time it does lend some credibility to the technique (Fig. 2). In addition, the use of the quadratic plateau polynomial regression model is unique to this study but presents a seemingly reliable way to determine the learning curve. This model allows a quadratic regression curve to be fit to the early data and a flat line (plateau) to the later data thus allowing the model to predict the exact point of plateau in the learning curve as it describes how many subjects were needed before the sonographer achieved a stable within-subject variance. Given four measurements per subject and a plateau at subject 21, this suggests that the error may plateau after approximately 80 individual measurements of ONSD.

Due to the difference in variance in early versus late subjects we questioned the validity of the measurements in the initial 21 subjects in our previous publication. However, despite the increased variability, since our original data was reported using the mean of four measurements any random measurement error should cancel out. By defining the early group as all subjects measured up to subject 21 and the late group as subjects 22–120 we compared both the mean measurements and the between-subject variance between these two groups. Neither of these revealed a difference confirming that despite the increased within-subject variance in the first 21 subjects, the overall mean and between subject variance was equal between these groups meaning the first 21 measurements were still valid.

### Study limitations

There are a number of significant limitations to this study performed in healthy volunteers. First, as mentioned earlier, when calculating the learning curve it is assumed that the actual ONSD measurement should be identical in each subject in both the right and left eye. However, even if these measurements are not identical [and our results would substantiate this since there still is some within-subject variability remaining even after the plateau (Fig. 2)] the plateau in this variability may still represent a useful definition of reliability. This is an area that requires further confirmation. Second, we have used a single operator to minimize inter-observer bias and hence to test the new statistical method for the estimation of the learning curve in a rather small group of healthy volunteers. This operator was not blinded to the measurements but the calculation of learning curve was a post hoc analysis and not the initial intention of the original study. Further studies are clearly required including in larger number of subjects and with more observers to confirm the current results. Finally, the operator in this study was in fact not a novice but his previous experience with this technique, although considerable, was on a more sporadic basis prior to this study. This does not lessen the finding of the learning curve but does suggest that the learning curve may be significantly longer than we have estimated in the case of true novices. In addition, it may also suggest a significant decay of skills if the technique is not practiced regularly and could suggest the need for retraining after a prolonged period of inactivity or regular maintenance of skills through simulation. The aforementioned concepts will be a focus of a future study.

## Conclusions

Despite the obvious limitations, this study offers a unique method of calculating the learning curve associated with ONSD utilizing a new definition of reliability by looking at within-subject variability and a unique statistical model to calculate the plateau in this variability.

## Abbreviations

ONSD: optic nerve sheath diameter
FAST: focused assessment with sonography in trauma
ICP: intra-cranial pressure
